# Poly(lactic acid)/Cellulose Films Produced from Composite Spheres Prepared by Emulsion-Solvent Evaporation Method

**DOI:** 10.3390/polym11010066

**Published:** 2019-01-04

**Authors:** Sónia Sousa, Ana Costa, Abílio Silva, Rogério Simões

**Affiliations:** 1 FibEnTech—Fiber Materials and Environmental Technologies Research Unit, Universidade da Beira Interior, Rua Marquês d’Ávila e Bolama, 6201-001 Covilhã, Portugal; anacosta@ubi.pt (A.C.); rmss@ubi.pt (R.S.); 2 C-MAST—Centre for Mechanical and Aerospace Science and Technology, Universidade da Beira Interior, Rua Marquês d’Ávila e Bolama, 6201-001 Covilhã, Portugal; abilio@ubi.pt

**Keywords:** poly(lactic acid), pulp fibers, biocomposite, emulsion-solvent evaporation method, films, mechanical properties

## Abstract

The compound of poly(lactic acid) (PLA) and cellulose was made by the emulsion-solvent evaporation technique in order to obtain spheres which are then compression molded to produce a biocomposite film. The effect of the dispersant (poly(vinyl alcohol)—PVA)/PLA ratio on the spheres yield was studied. Moreover, to evaluate the effect of cellulose particle size and surface chemistry on the process yield, unbleached eucalypt kraft pulp and microcrystalline cellulose (MCC), both unmodified and physically or chemically modified were used. PLA/cellulose spheres were characterized regarding its physical properties. It was found that the spheres yield is essentially determined by the PVA/PLA ratio and the percentage of cellulose incorporation is greatly affected by the surface chemistry of cellulose. Regarding the films, DSC runs showed a significant effect of the cellulose type incorporated into PLA matrix on the cold crystallization temperature and on the degree of crystallinity of the biocomposite films. The measurement of tensile properties of the biocomposite films revealed that the strength, elongation at break and toughness (tensile energy absorption at break) of the films incorporating unmodified and chemically modified MCC were substantially improved.

## 1. Introduction

In the packaging industry, plastic materials are customizable, easy to process and cheap. The great majority of plastics materials are made up from non-renewable petroleum-based fossil fuels. Due to limited nature of fossil fuels and the growing environmental concern on their use, there is a need for finding more environmentally friendly materials. Currently, the development of biodegradable polymer composites combining biodegradable matrices and natural fibers as reinforcement is of great importance [1]. Starch, poly(caprolactone) (PCL), poly(lactic acid), poly(butylene succinate) (PBS) and poly(hydroxyalkanoates) (PHAs) are most commonly used as matrix phase biopolymers [1,2,3,4,5,6]. Among the polymer matrices, PLA is one of the most used and at present PLA-based materials find applications in the biomedical, textile and packaging areas [5,7]. PLA is a high molecular weight polyester usually produced by ring-opening polymerization of lactide. The monomer, lactic acid, can be derived from corn, potato, cane, beet sugar or cheese residues by fermentation [1,3,7]. The commercial PLA, however, is a brittle material and various reinforcing materials such as natural fibers can be used to improve its toughness [8,9]. In addition, natural fibers as reinforcement materials present other advantages as their low density, high specific strength and modulus, nonabrasive character and low cost [3]. Various forms of cellulose have been explored as reinforcements into PLA composites. These include fibers from jute, kenaf, henequen, flax, cotton, bamboo [10,11,12,13,14,15,16], wood pulp [3,4,17], microcrystalline cellulose (MCC) [9,18,19] and, micro and nanocellulose in different forms and from different origins [8,20,21,22,23,24,25,26,27,28]. Due to its hydrophilic nature, cellulose is not easily uniformly dispersed into hydrophobic PLA matrix which leads to poor interfacial adhesion, thus reducing the mechanical properties of the composites [16,18,22,23]. To overcome this limitation, many physical or chemical treatments have been applied for cellulose modification. The effect of plasma treatment on interfacial interaction of lyocell fibers in a poly(lactic acid) matrix was investigated [29]. According to Cho et al. [10], natural fibers treated with tap water by static soaking and dynamic ultrasonication, followed by drying leads to PLA composites with improved interfacial shear strength, flexural and dynamic mechanical properties. Treatment of cellulosic fibers with alkali or enzymes were also tested [12,13]. Incorporation of casein protein or bioadimide in cellulose/PLA composites was found to improve the dispersion and fiber/matrix adhesion [15,30]. In addition, Magniez et al. [16] demonstrated that the adsorption of amphiphilic poly(ethylene glycol)-b-poly(l-lactide) block copolymers increases the interfacial affinity in a jute and polylactide biocomposite. Methods such as acetylation, silylation or grafting with other hydrophobic monomers have been used for cellulose surface modification [6,11,20,31]. The preparation of microcrystalline cellulose-graft-poly(lactic acid) via melt copolycondensation was carried out by Hua et al. [18]. These authors observed that the microcrystalline cellulose grafting increases the compatibility with PLA matrix thus improving the dispersion of the cellulose. Graupner [12] and Graupner et al. [32] found that the use of lignin in PLA/cellulose fiber composites improved the adhesion between fiber and matrix. Recently, nanofibrillated cellulose with high lignin content was used to enhance the PLA/cellulose compatibility in the composites [33,34].

In addition to cellulose chemical properties and its dispersion in the PLA matrix, the biocomposite properties are also affected by the processing conditions. Melt-compounding is traditionally used for many types of biocomposites as it is fast and easier to scale-up to industrial scale [35]. However, as PLA is sensitive to temperature, shearing and hydrolysis, some precautions are required during the processing step to avoid its degradation [7]. Solvent casting is a simple and widely used technique to prepare cellulose nanocomposites using different matrices, but it is mostly applied at laboratory scale [7,25,30,35]. This technique can lead to differences in the properties of the composites compared to another processing methods [7,36]. In another approach, a papermaking based process was used, which enables the production of sheets made from aqueous suspension containing a mixture of cellulose fibers or microfibrillated cellulose (MFC) and PLA fibers [8,37]. This process gives good dispersions even at high MFC contents (up to 90 wt %) and the composites obtained by compression molding of the stacked sheets presented an increase of tensile properties with the MFC content [8]. The use of PLA microparticles is an alternative to PLA fibers resulting in nanocomposites with good cellulose nanofibers dispersions [20,22]. The production of PLA microparticles was carried out by solvent evaporation technique which is widely used in pharmaceutical industry for drug microencapsulation [22,38,39]. In a typical procedure, a volatile organic phase containing the dissolved polymers is emulsified in an aqueous phase at continuous constant stirring rate. Then, the volatile solvent is gradually evaporated to form the spherical polymer particles [39]. This technique was also used by Lee and Ji [40] to encapsulate hydrophilic entities such as cellulose nanowhiskers and microfibrils within polymeric nano/microspheres. However, to the best of our knowledge, until now the solvent evaporation technique was never used to produce PLA spheres incorporating pulp fibers, which have a much higher size. In another study, Lee et al. [26] developed a method based on thermally induced phase separation to produce composite microspheres of bacterial cellulose with PLA that can be processed in conventional extrusion.

The aim of this work is to produce biocomposite spheres of PLA and both pulp fibers and MCC, using emulsion-solvent evaporation method. In order to evaluate the effect of size and surface properties of the pulp fibers, unbleached pulp fibers and the corresponding grinded fibers, either unmodified or surface modified by lignin deposition were used and the effectiveness of cellulose incorporation into PLA spheres was analyzed. MCC and MCC surface modified were also studied. Spheres of the PLA/cellulose biocomposite were characterized for its physical and chemical properties. The produced spheres are processed by compression molding to obtain composite films, which are characterized regarding its thermal, structural and mechanical properties.

## 2. Materials and Methods

### 2.1. Materials

PLA used in this study was Ingeo 2500HP from NatureWorks LLC (Minnetonka, MN, USA) having a density of 1.24 g/cm^3^ and relative viscosity of 4.0 (tested at 1.0 g/dL in chloroform). Microcrystalline cellulose (MCC) was Avicell^®^ purchased from Merck (Darmstadt, Germany). Hexadecyltrimethylammonium bromide (CTAB) and poly(vinyl alcohol) (PVA, 98–99% hydrolysis, *M*_w_ = 85,000–124,000) were purchase from Sigma-Aldrich (St. Louis, MO, USA). Dichloromethane (DCM) (Scharlau, Sentmenat, Spain), hydrochloric acid (Merck, Darmstadt, Germany), sulfuric acid (Sigma-Aldrich, USA), potassium permanganate (Merck, Darmstadt, Germany), potassium iodide (VWR, Leuven, Belgium) and sodium thiosulfate pentahydrate (Merck, Darmstadt, Germany) were analytical grade and used as received. The carbohydrate standards d-glucose and d(+)-xylose were acquired from Pronalab (Sintra, Portugal) and Merck (Darmstadt, Germany), respectively. Cellulosic fibers from *Eucalyptus globulus* unbleached kraft pulp and kraft black liquor were supplied by Celtejo—Empresa de Celulose do Tejo, S.A (Vila Velha de Ródão, Portugal). Kraft black liquor was used as a source of lignin for fiber surface modification. Their organic and inorganic content were 12.7% and 14.4%, respectively, determined using the standard methods TAPPI 650 om-09 and TAPPI T211 om-02.

### 2.2. Cellulose Modification

The average size of the pulp fibers was modified using the mixer mill CryoMill (Retsch, Haan, Germany). For that, 2.5 g of unbleached pulp fibers (UP) were grinded at room temperature using one grinding cycle of 2.5 min at 30 s^−1^ frequency. This sample will be designated by GUP. The fiber morphology of UP and GUP samples were characterized using the fiber analyzer Morfi (Techpap, Grenoble, France). The arithmetic average fiber length (mm), width (µm) and fines content (% in length) of UP were 0.641, 18.7 and 28.7, whereas the corresponding values for GUP were 0.447, 20.9 and 62.9. Particles with less than 0.2 mm in length are considered as fines [41]. The mean particle size of MCC was 13 µm, determined by laser diffraction particle size analyzer (Beckman Coulter, Inc., Brea, CA, USA).

In order to modify the surface chemistry of the cellulose, kraft lignin was precipitated over the MCC and the grinded pulp fibers as follows: 2.5 g of cellulose in aqueous suspension (50 mL deionized water) was mixed with 50 mL of black liquor. Then the pH of the cellulose-lignin was lowered by addition of 5 M HCl solution, until pH around 3. The suspension was centrifuged at 3000× *g* for 10 min, the supernatant removed and the cellulose modified with lignin was washed three times with deionized water. In order to obtain cellulose samples with different lignin content, an additional assay was carried out using 2.5 g of GUP suspended in 90 mL deionized water and 10 mL of black liquor. These samples were designated as MCC-L61, GUP-L61 and GUP-L25, respectively, where L61 and L25 means the lignin content in the modified cellulosic materials. The lignin content was determined according to TAPPI T236 om-99 standard and was found to be 4.1%, 25.4%, 60.9% and 61.4% for GUP, GUP-L25, GUP-L61 and MCC-L61 samples, respectively.

### 2.3. Preparation and Characterization of PLA/Cellulose Biocomposite Spheres

**Figure 1:**
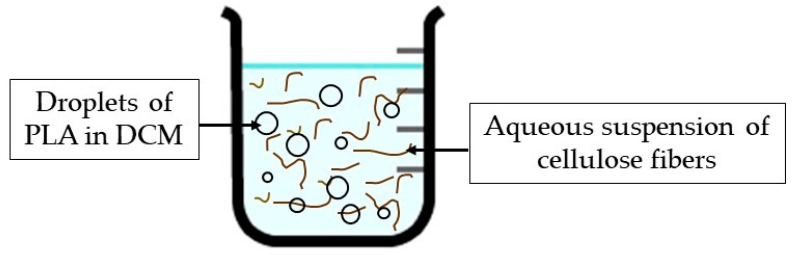
Illustration to show the oil-in-water emulsion in the process to obtain the PLA/cellulose biocomposite spheres.

In a typical laboratorial procedure, a suitable amount of PVA was dissolved in 110 mL deionized water containing 0.1 g CTAB and stirred at 300 rpm using a mechanical stirrer (Heidolph RZR 2021, Schwabach, Germany). Separately, 0.5 g cellulose fibers was suspended in 50 mL of deionized water using an IKA^®^ T25 UltraTurrax^®^ homogenizer at 10,000 rpm for 1 min, and after this suspension was added to the PVA solution. After homogenization of the PVA and the cellulose fibers, the solution of 2 g PLA in 40 mL dichloromethane (previously prepared) was poured into the aqueous suspension producing an oil-in-water emulsion (Figure 1). PVA is commonly used in emulsification solvent evaporation formulations as dispersant to prevent the coalescence of the oil droplets, having been observed that the used amount affects the preparation of microspheres [38,39,42]. Since the cellulose pulp has carboxylic groups, a cationic surfactant (CTAB) was used together with PVA to stabilize the emulsion and to promote cellulose incorporation in the PLA. The amount of CTAB was kept constant and PVA concentration was changed in order to evaluate its effect on the process yield. The system was mechanically stirred at constant rate (300 rpm) for 24 h at room temperature (20 °C) to evaporate the organic solvent. The spheres were recovered from the liquid phase by sedimentation, thoroughly washed with deionized water in order to remove the dispersants (PVA and CTAB) and dried at 40 °C for 2 days. An assay without cellulose addition was also carried out using the same procedure, and the PLA spheres were recovered.

#### 2.3.1. Process Yield

The spheres yield was determined by the ratio of the weight of spheres and the sum of the weight of PLA and cellulose charged in the assay, assuming that the entire amount of dispersants (PVA and CTAB) remains in the liquid phase (supernatant). The amount of cellulose and lignin incorporated in the biocomposite spheres was estimated by mass balance, after measuring their corresponding amounts remaining in the liquid phase after solid separation. Therefore, the percentage of cellulose and lignin incorporation into PLA spheres was calculated by the difference between the initial amount of cellulose and lignin used in each assay and the amount of cellulose and lignin in the liquid phase, regarding the initial charge. The cellulose and lignin in the liquid phase were determined following the standard procedure usual in the wood and pulp filed. For cellulose determination, the NREL procedure (NREL/TP-510-42618) was followed. It should be noted that cellulose term used throughout the work also includes the residual hemicellulose in the pulp fibers. The dissolved lignin in the liquid phase was determined following the TAPPI T236 om-99 standard procedure; a blank test was carried out using the same proportions of PVA and CTAB as those used in the corresponding assay.

#### 2.3.2. Physical Characterization of PLA/Cellulose Spheres

The particle size distributions and external specific surface area of the most representative samples of PLA/cellulose spheres was obtained by the laser diffraction method using the Coulter LS200 particle size analyzer (Beckman Coulter Inc., Brea, CA, USA). The density of the spheres was measured by the pycnometer method. Global specific surface area was measured according to the Brunauer-Emmett-Teller (BET) method using ASAP 2000 V2.04 (Micromeritics Instruments Corp., Norcross, GA, USA) apparatus. To further evaluate the morphology of the biocomposite spheres, scanning electron micrographs were taken on a Hitachi S-2700 scanning electron microscope (Tokyo, Japan) at 20 kV accelerating voltage. A cross-section of the spheres was obtained by imbedding the spheres into melted paraffin. After complete solidification, the hardened paraffin was cut into pieces using a Microm HM 315 rotary microtome and the sphere slices were washed with xylene. The samples were coated with a gold layer allowing for surface and cross-section visualization.

#### 2.3.3. Attenuated Total Reflectance-Fourier Transform Infrared Spectroscopy

The efficiency of chemical surface modification was analyzed by ATR-FTIR spectroscopy. The ATR-FTIR spectra have been recorded on a Thermo Scientific Nicolet iS10 spectrometer (Thermo Fisher Scientific, Waltham, MA, USA) equipped with an ATR unit. The cellulose samples and the PLA/cellulose biocomposite spheres were analyzed in the form of pellets. A total of 64 scans were performed over each sample with a resolution of 4 cm^−1^. The spectrum was obtained from a range of 600–4000 cm^−1^.

#### 2.3.4. Contact Angle Measurements

In order to determine the changes in the hydrophilic character of the cellulose after surface modification, as well as the hydrophilicity of the PLA/cellulose composite spheres, contact angle measurements were carried out with deionized water using a DataPhysics OCAH 200 system (DataPhysics Instruments GmbH, Filderstadt, Germany) by means of sessile drop method. Uniform and practically porosity-free pellets (obtained by high compression at room temperature) of cellulose and lignin samples and PLA/cellulose spheres were used for this purpose. The initial contact angle was measured after ca. 50 ms of the water drop fall on the pellet surface. This time may be considered short enough to avoid absorption and thus an equilibrium drop shape could be assumed. All measurements were performed at 22 °C and each sample was measured with a minimum of four drops.

### 2.4. Production and Characterization of Biocomposite Films from PLA/Cellulose Spheres

The produced biocomposite PLA/cellulose spheres were compression molded into films using a hot press at 180 °C, progressively increasing the compression pressure to 3 MPa for 1 min. This temperature and pressure were kept constant for 1 min. Then, the heating was turned off and the hot press was cooled down to 110 °C at a rate of 6 °C/min using a circulating bath. The pressure was relieved and the film taken out.

#### 2.4.1. Differential Scanning Calorimetry Analysis

Thermal properties of the PLA and biocomposite films were analyzed by Differential Scanning Calorimetry (DSC). The analyses were conducted with a Netzsch instrument DSC200 Cell and TASC 414/3 controller (Netzsch-Gerätebau GmbH, Selb, Germany). Samples of about 10 mg were placed in aluminum pans and heated from 30 to 500 °C at 10 °C/min using nitrogen as a purge gas. The degree of crystallinity (*X_c_*) of the film samples was calculated by [43]:

(1) $$X_{c}\left(\%\right)=\frac{\left(\Delta H_{m}-\Delta H_{cc}\right)}{\Delta H_{m}^{0}\cdot w}\cdot 100$$

where $\Delta H_{m}$ and $\Delta H_{m}^{0}$ is the melting enthalpy of the sample and of the pure crystalline PLA (93.6 J/g) [25], respectively; $\Delta H_{cc}$ is the cold crystallization enthalpy and *w* is the weight fraction of PLA in the biocomposite.

#### 2.4.2. Structural Properties of the Biocomposite Films

Basis weight was determined by the ratio between the weight and the area of the films according to ISO 536:2012. The films were weighed in an analytical balance (±0.0001 g). The thickness of the films was measured according to ISO 534:2011 using the micrometer Adamel Lhomargy model MI 20 (Ivry-sur-Seine, France). Apparent density of the biocomposite films corresponds to the ratio between the basis weight and the thickness, according to ISO 534:2011. Structural properties were evaluated at 23 °C and 50% RH.

#### 2.4.3. Mechanical Testing of Biocomposite Films

Tensile tests were performed on a tensile tester Thwing-Albert Instrument Co. (West Berlin, NJ, USA), EJA series based on ISO 1924/2:2008 standard. The biocomposite films were cut into rectangular pieces with 10 mm wide and 40 mm length, and then subjected to tensile test at a strain rate of 10 mm/min, using the initial grip distance of 10 mm. Tensile strength, elongation at break, Young’s modulus and toughness (tensile energy absorption at break) were calculated. Bending tests were accomplished on a Lorentzen and Wettre stiffness tester (Stockholm, Sweden) at bending angle of 15°, based on ISO 2493/1:2010 standard and the bending elastic modulus was calculated according to Niskanen [44]. All tests were performed at 23 °C and 50% RH.

## 3. Results and Discussion

### 3.1. Spheres Yield and Effectiveness of Cellulose Incorporation

**Figure 2:**
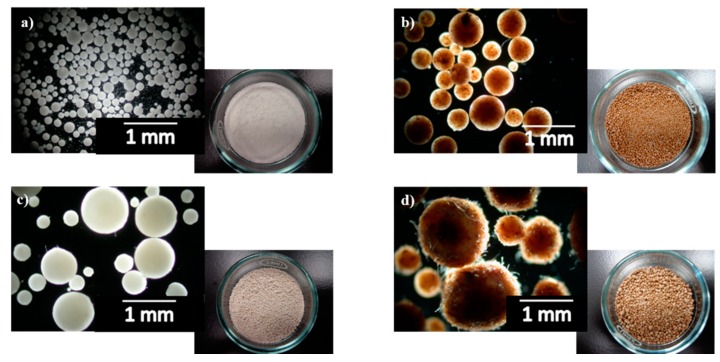
Pictures of some PLA/cellulose biocomposite spheres: (**a**) PLA/MCC; (**b**) PLA/MCC-L61; (**c**) PLA/GUP; (**d**) PLA/GUP-L61 (see Table 1 for sample identification).

PLA/cellulose biocomposite spheres were successfully obtained (as seen in Figure 2) by solvent evaporation technique with yields ranging from close to 0 to 87.2% (Table 1); this yield represents the percentage of the PLA and cellulose (jointly accounted) recovered as spheres regarding to the initial amounts used.

As observed in Table 1, the PVA/PLA ratio determines the spheres yield, as can be seen for the PLA samples with PVA/PLA ratios of 0.13, 0.28 and 0.35, the PLA/GUP-L61 samples obtained with 0.13, 0.20 and 0.28 PVA/PLA ratios, and the PLA/MCC samples with 0.13, 0.20 and 0.28 PVA/PLA ratios. When only PLA was used, the increase of PVA/PLA ratio from 0.13 to 0.35 leads to the increase of spheres yield from 36.4% to 93.2%. For the low PVA/PLA ratio, a significant spheres agglomeration occurs. Increasing the amount of PVA stabilize the emulsion, prevents the droplets coalescence during the dichloromethane evaporation by providing a protective layer around the droplets. Thus, the droplets aggregation into large particles does not occur and the spheres yield increase with higher PVA/PLA ratio. Jiang et al. [42] also observed an increase of PLA nanoparticles yield with the increase of PVA concentration. When the emulsion contains cellulose fibers (original or grinded) besides PLA, the PVA/PLA ratio required to form individualized spheres is lower than those required by PLA only (0.13 versus 0.35); however the average spheres size is much higher and the external specific surface area much lower (Table 2). Reducing cellulose fiber length from 0.641 mm (UP) to 0.447 mm (GUP) with the concomitant increase of the cellulose fine material content (from 28.7% to 62.9%), the spheres yield increased only slightly from 79.9% to 82.8% (PLA/UP vs. PLA/GUP), which suggest that the particle dimensions are not a key parameter in spheres yield. In addition, the increase of PVA/PLA ratio from 0.13 to 0.20 (PLA/GUP) does not improve the spheres yield. In the case of MCC, the amount of dispersant required is higher than for the cellulose fibers. For PVA/PLA ratios of 0.13 or 0.20, the formation of agglomerates of irregular size occurred, providing a very low spheres yield (around 4%), whereas with the 0.28 PVA/PLA ratio a spheres yield of 74.0% was obtained. This high amount of PVA required for spheres formation is in accordance with the very high external specific surface area of these particles (Table 2). In addition, as we will see later on, the MCC incorporation is very low and therefore the droplets are almost only PLA; as a consequence, the PVA/PLA ratios were also of the same magnitude.

**Figure 3:**
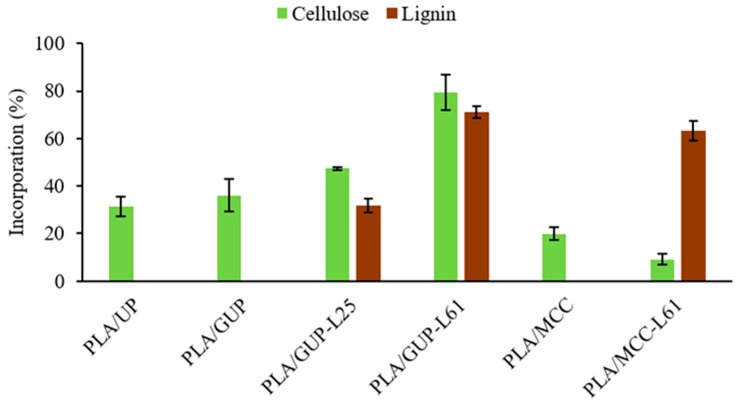
Cellulose and lignin incorporation (based on the corresponding amounts initially charged) into the PLA spheres (see Table 1 for abbreviations).

In addition to spheres yield, it is important to investigate the cellulose material incorporation into the spheres composite. The PLA:cellulose ratio initially charged into the medium was fixed at 80:20. The cellulose incorporation was calculated by mass balance (charged cellulose—cellulose recovered in the liquid phase after solid/spheres recover), regarding the cellulose charged, and the corresponding values are in Figure 3.

Grinding the cellulose fibers led to a small increase in incorporation (from 31.4% to 36.0%, Figure 3), which suggests that the particle dimension is not the main parameter determining the cellulose fibers incorporation. According to Maa and Hsu [45] and Al-Azzam et al. [46], the incorporation of solid protein particles (0.4–79 µm particle sizes) in poly(metyl methacrylate) and poly(lactic-co-glycolic acid) polymers was improved with decreasing protein particle size. It was also found that the protein particle with irregular shape resulted in microspheres with lower amount of protein than if protein particles are spherical, for protein particle size higher than 5 μm. For finer particle, the effect was less significant [45]. In the present work, the UP and GUP fibers have irregular shape and their sizes (641 and 447 µm) are much higher than the protein particle sizes used in the abovementioned work (0.4–79 µm). Considering their size, the behavior of microcrystalline cellulose (MCC) is at first glance unexpected, because the lowest incorporation was observed despite its lowest average particle size (13 µm). This result demonstrates the low affinity of pure cellulose to the PLA (dissolved in dichloromethane). On the contrary, the unbleached pulp fibers (UP and GUP) even with a very small amount of lignin (4.1%), exhibit higher affinity to PLA. These results observed in the emulsion are in agreement with those reported for the solid phase. In fact, several authors [10,32,33], stated that lignin can be used as coupling agent in cellulose fiber reinforced composites as it improves chemical interactions between the cellulose fiber and PLA matrix through non-polar groups.

Therefore, the other approach exploited in this work was the cellulose fibers surface modification, using a natural product, kraft lignin. Kraft lignin is a sub-product of the kraft pulp production process, easily removed from the black liquor (a side stream of the process) by acidification. In wood, lignin has an important hydrophobic character; in the kraft process it is partially depolymerized and chemically modified in order to make it soluble in strong alkaline medium. By acidification it turns again water insoluble and can be adsorbed on the fibers. This lignin, however, is not hydrophobic according to the standard definition (surface having a water contact angle greater than 90°), as the water contact angle measured in the lignin pellets was around 57°. Nevertheless, this value is higher than those measured on MCC (41.2°) and unbleached pulp pellets (48.3°).

In order to investigate the effect of this surface modification on both the spheres yield and cellulose incorporation into PLA spheres, MCC and grinded pulp fibers (GUP) with precipitated lignin were prepared. Comparing GUP (without precipitated lignin) and GUP-L61 (with precipitated lignin) samples in Table 1, it was observed that for the pulp fibers with 61% lignin (GUP-L61), the PVA/PLA ratio should be increased from 0.13 to 0.28 in order to obtain individualized spheres with a yield of 87.2%. For a PVA/PLA ratio of 0.13 or 0.20, practically only formation of agglomerates occurred. A possible explanation for the higher PVA/PLA ratio required may be the more hydrophobic nature of lignin; PLA alone also requires high PVA/PLA ratio. During the dichloromethane evaporation, the coalescence of the droplets may occur by hydrophobic affinity, leading to a higher amount of PVA required to prevent the aggregation of the GUP with adsorbed lignin. Concerning to the MCC, unmodified and modified with 61% lignin (MCC vs. MCC-L61), it was found that the presence of lignin decreases the PVA/PLA ratio required for the formation of individualized spheres from 0.28 to 0.13, with a spheres yield of 84.3%, but the average spheres size (weighted in volume) increased from 131 to 642 µm (Table 2) and the corresponding external specific surface area decreased from 580 to 101 cm^2^/g. Considering that the GUP-L61 sample had required a PVA/PLA of 0.28, it was not expected that the PVA/PLA ratio of 0.13 would be enough for MCC-L61 sample, even because the external specific surface areas of the two samples are of the same magnitude. As it is well known in paper technology that the cohesion energy of fiber flocks increases with fiber length, we can speculate that higher PVA/PLA ratio is required to counterbalance the cohesion energy of the long fibers, regarding the MCC particles (447 µm vs. 13 µm).

Regarding cellulose fibers incorporation (Figure 3), for the GUP fibers it was observed an increase of cellulose incorporation from 36.0% to 79.4% with the increase of lignin content from 4% to 61%. Moreover, the lignin retention exhibits a similar trend. In other words, the pulp fiber incorporation in the PLA spheres increases from 36% to over 70%, with the increase of the fiber lignin content. For the MCC, the behavior is the opposite; there is a decrease when the cellulose is modified with lignin (Figure 3). This result may be due to desorption of the previously precipitated lignin on the MCC, by preferential dissolution in dichloromethane. On the other hand, the high lignin incorporation (63.1%) in the PLA spheres is a clear indication of the high affinity of the lignin to the PLA (see, also Table 3). 

Based on the amount of cellulose and lignin charged and the corresponding incorporation, the content of each component in the composite spheres was calculated and the values are presented in Table 3. It was observed that the cellulose content in the spheres ranged from 1.0 to 8.8%, with pulp fibers in clear advantage.

### 3.2. Physical Characterization of PLA/Cellulose Spheres

**Figure 4:**
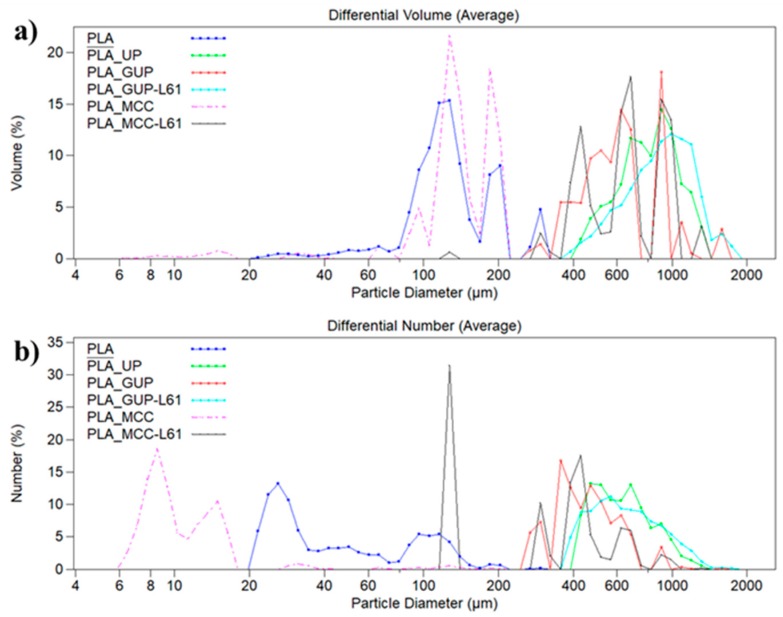
Particle size distributions in volume (**a**) and in number (**b**) for some PLA/cellulose biocomposite spheres (see Table 1 for abbreviations).

Some of the most representative samples of PLA/cellulose biocomposite spheres (indicated in Table 1 by asterisks) were characterized concern to physical properties, namely density, particle size distributions and external specific surface area (Table 2). All samples presented multimodal particle size distributions (Figure 4). PLA spheres size range between 20 and 325 µm with low average size (126.2 µm and 46.0 µm, respectively weighted in volume and number) with a high external specific surface area of 525 cm^2^/g (Table 2). The incorporation of unbleached pulp (PLA/UP) provides particles size ranging from 390 to 1400 µm with an average size of 796.5 µm (volume weighted) and 633.4 µm (number weighted), close to the magnitude of the fiber length. The external specific surface area of these spheres, determined by laser diffraction technique, was 78.3 cm^2^/g. The grinding of the pulp fibers (PLA/GUP sample) results in spheres with smaller size (average size volume weighted of 611.1 µm and number weighted of 447.2 µm) than PLA/UP spheres. The lower average size provides a higher external specific surface area for the PLA/GUP spheres compared with original unbleached pulp (PLA/UP) (104.5 cm^2^/g versus 78.3 cm^2^/g). Thus, it can be concluded that fibers with lower length (0.447 mm) yields smaller spheres than the corresponding original fibers (average fiber length of 0.641 mm). As reported in Table 2, the incorporation of grinded pulp fibers modified by lignin precipitation produces spheres with the largest average size (888.0 µm and 648.4 µm, respectively weighted in volume and number) with a size distribution ranging from 370 to 1900 µm. Notice that these spheres have average size higher than those obtained by incorporation of original pulp fibers. Moreover, they also present the lowest external specific surface area (71.1 cm^2^/g). Thus, the spheres size is not only affected by the size of the incorporated fibers but also by their surface chemistry. PLA/MCC spheres differ from the others biocomposite spheres by having the smallest size weighted in volume and in number (130.9 µm and 10.9 µm, respectively). The modification of MCC with lignin leads to spheres with much higher size than MCC (average size increases from 130.9 to 641.7 µm, weighted in volume, and from 10.9 to 305.5 µm weighted in number) and lower external specific surface area (101.0 cm^2^/g).

**Figure 5:**
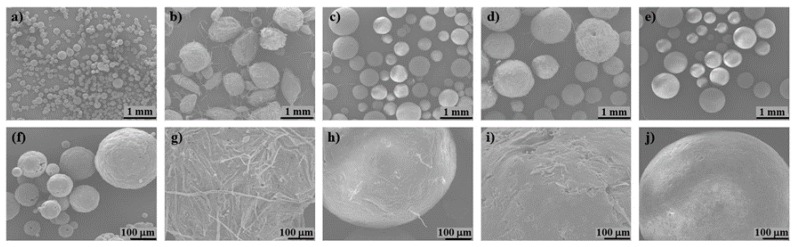
SEM images of the surface morphology of biocomposite spheres: (**a**,**f**) PLA; (**b**,**g**) PLA/UP); (**c**,**h**) PLA/GUP; (**d**,**i**) PLA/GUP-L61; (**e**,**j**) PLA/MCC-L61; (**a**) 200× and (**f**) 1000× magnification; (**b**–**e**) 25× and (**g**–**j**) 200× magnification (see Table 1 for sample identification).

**Figure 6:**
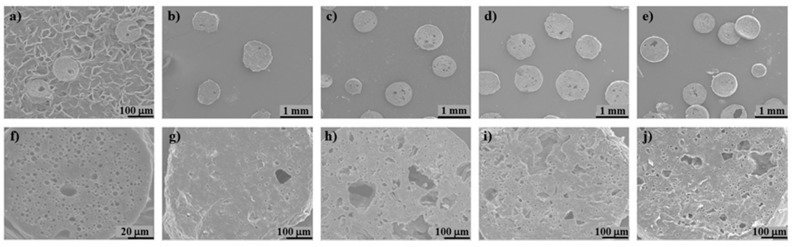
SEM images of the inner structure of biocomposite spheres: (**a**,**f**) PLA; (**b**,**g**) PLA/UP; (**c**,**h**) PLA/GUP; (**d**,**i**) PLA/GUP-L61; (**e**,**j**) PLA/MCC-L61; (**a**) 200× and (**f**) 1000× magnification; (**b**–**e**) 25× and (**g**–**j**) 200× magnification (see Table 1 for sample identification).

The global (external and internal) specific surface area provided by BET analysis is one or two orders of magnitude higher than those obtained by laser diffraction. This is an expected result due to the surface roughness and the internal porosity, accounted by BET analysis, and also revealed by the SEM images (Figure 5 and Figure 6). The PLA/UP sample exhibits a high roughness due to the presence of the larger fibers, whereas others such as PLA and PLA/MCC-L61 are much smoother. All the biocomposite spheres are spherical except in the case of PLA/UP sample. The cross-sections of the spheres (Figure 6) show that the inner structure has some pores, presenting the PLA/UP sample a more compact structure. The internal porosity observed in Figure 6 is in good agreement with the spheres density (0.69–0.91 g/cm^3^, Table 2), which is significantly lower than the 1.24 g/cm^3^ reported for the PLA used in the present study.

### 3.3. Chemical Characterization of PLA/Cellulose Spheres

**Figure 7:**
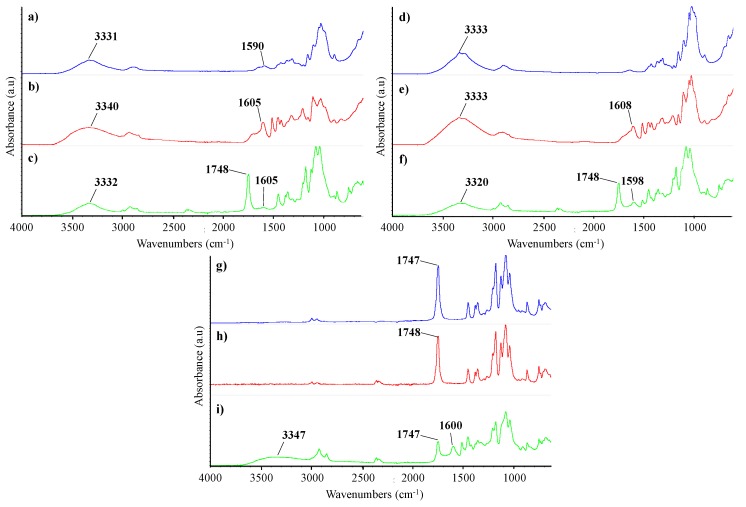
ATR-FTIR spectra of cellulose samples, PLA and biocomposite spheres: (**a**) UP or GUP; (**b**) GUP-L61; (**c**) PLA/UP; (**d**) MCC; (**e**) MCC-L61; (**f**) PLA/GUP-L61; (**g**) PLA; (**h**) PLA/MCC; (**i**) PLA/MCC-L61 (see Table 1 for abbreviations).

Surface chemistry induced by the chemical modifications of the cellulose samples as well as of the PLA/cellulose biocomposite spheres were investigated by ATR-FTIR (spectra in Figure 7). When compared with MCC, ATR-FTIR spectrum of unbleached pulp fibers showed an additional band at 1590 cm^−1^ characteristic of lignin which is associated to aromatic ring vibration [32,47]. Both spectra exhibit the typical bands of cellulose, namely the broad band at 3331–3333 cm^−1^ attributed to the OH stretching vibration of intramolecular hydrogen bonds [32,48]. The spectra of GUP and MCC modified with precipitated lignin (GUP-L61 and MCC-L61) showed new peaks (at 1605 and 1608 cm^−1^, respectively) comparatively to GUP and MCC spectra, assigned to lignin [47,48], confirming the lignin adsorption. PLA spectrum showed distinctive absorption bands assigned to their different functional groups, corresponding the major peak at 1748 cm^−1^ to the C=O stretching vibration [49]. The biocomposite spheres spectra confirm the presence of lignin (peak around 1598–1605 cm^−1^), cellulose (for example, the peak around 3331–3347 cm^−1^) and PLA (for example the peak around 1707–1708 cm^−1^). The PLA/MCC spectrum is very similar to that of PLA due to the very low incorporation of MCC into the spheres (these are constituted by 95% PLA). Comparing the spectra of the cellulose samples and PLA/cellulose biocomposites, it was observed a decrease in the intensity of the peak assigned to OH stretching vibration (around 3331–3347 cm^−1^) for the biocomposites, as expected. Compared to the PLA, the wavenumber of C=O peak in the biocomposites remains practically unchanged (1748 vs. 1747 cm^−1^ for PLA/MCC-L61 sample) suggesting a relatively weak interaction between cellulose (UP, GUP-L61 and MCC-L61) and PLA.

**Figure 8:**
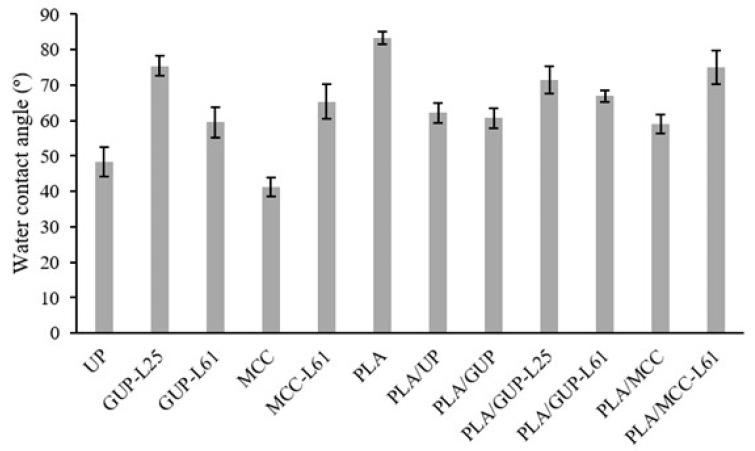
Water contact angle of cellulose samples, PLA and PLA/cellulose biocomposite spheres (see Table 1 for abbreviations).

Figure 8 shows the water contact angle for the starting materials and the composites spheres as well, measured in pellets produced under high pressure (0.9 GPa) at room temperature in order to preserve the chemistry and minimize the porosity and roughness of the pellet which both affect contact angle measurement. By this way, the contact angle reflects mainly the chemistry of the material. MCC presents the lowest water contact angle (41.2°), which is the most hydrophilic sample. In turn, unbleached pulp presents a higher contact angle (48.3°) due to the presence of residual lignin. As expected, the lignin precipitation on the cellulose fibers and MCC increases very significantly the water contact angle. For the unbleached pulp, the contact angle increases from 48.3° to 75.4° (GUP-L25; around 25% lignin) and then decreases to 59.6° (GUP-L61; around 61% lignin). This decrease from 75.4° to 59.6° is probably due to the co-precipitation of sugar-lignin complex for the more concentrated black liquor solution. In fact, the lignin precipitated from the more diluted solution exhibited a darker tone than that precipitated from the more concentrated solution, despite the same final pH value used (pH = 3). Comparing the unbleached fibers and MCC, before and after lignin precipitation, we can observe a very significant increase of the water contact angle, although none samples have reached hydrophobic character (water contact angle higher than 90°), they have a contact angle value much closer to that of PLA which is 83.3°, consistent with those found in literature [50,51]. Thus, it would be expected that chemically modified cellulose samples were more compatible with PLA than those unmodified, resulting in higher percentage of cellulose incorporation (Figure 3). This behavior was in fact confirmed for GUP-L25 and GUP-L61, pulp fibers. For the microcrystalline cellulose reversible lignin desorption in aqueous medium was reported by Maximova et al. [52], which render the MCC-L61 sample hydrophilic and with low affinity to PLA; on the contrary, the desorbed lignin was strongly retained in the PLA (see Figure 3 and Table 3). Regarding the contact angles for the PLA/cellulose biocomposite spheres, the values are always inferior to that of PLA, ranging from 59.0° to 75.0° for PLA/MCC and PLA/MCC-L61 samples, respectively. Overall, the cellulose samples with higher contact angle leads to the corresponding PLA/cellulose spheres with slightly higher contact angle.

### 3.4. Characterization of the Biocomposite Films

**Figure 9:**
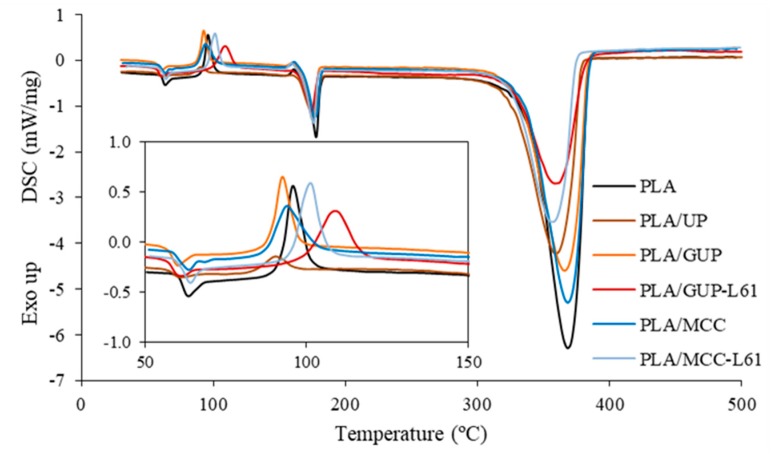
DSC curves of PLA and biocomposite films with the crystallization region in the insert (see Table 1 for abbreviations and Table 3 for composite composition).

Composite spheres were compression molded into films and these were characterized concerning their thermal, structural and mechanical properties. Thermal properties were investigated via DSC. The DSC curves are depicted in Figure 9 and were used to determine the glass transition temperature (*T_g_*), cold crystallization temperature (*T_cc_*), cold crystallization enthalpy (Δ*H_cc_*), melting temperature (*T_m_*), melting enthalpy (Δ*H_m_*) and degradation temperature (*T_d_*) of the different samples. At the end of the DSC test, the aluminum pans were weighed in order to determine the solid residue at 500 °C. These results are shown in Table 4 along with the degree of crystallinity (*X_c_*). It can be seen that the PLA exhibited a *T_g_* at 62.1 °C and the composite films have a slightly lower *T_g_*, except the PLA/MCC-L61 film (1% cellulose) that presented similar *T_g_* to the PLA. The decrease of the *T_g_* signifies that cellulose incorporation increases the chain mobility of the PLA polymer. It was also observed that the PLA fraction undertaken cold crystallization in the DSC test is composite dependent. For example, the original cellulose fiber (UP) led to the smallest cold crystallization peak (Figure 9), but the film exhibits the highest PLA crystallinity degree (Table 4). This indicates that this composite had favorable conditions to crystallize in the film making process. The contrary occurred for the composite incorporating grinded UP (GUP). Compared with PLA, the cold peaks shifts to lower temperatures for PLA/UP, PLA/GUP and PLA/MCC composites, indicating that cold crystallization occurs earlier induced by UP, GUP and MCC which act as nucleating agents for PLA crystallization, in good agreement with results reported by other authors [26,43,53,54]. On the contrary, the presence of lignin in the cellulose materials (PLA/GUP-L61 and PLA/MCC-L61) led to higher *T_cc_*. This result is in agreement with reported by Gordobil et al. [50] which found that the introduction of lignin in the PLA matrix affects the crystallization behavior causing that the PLA chains have a lower mobility and crystallize with greater difficult and higher temperatures. According to the reported by Graupner [12] and Graupner et al. [32], lignin improves the fiber/matrix adhesion, which may explain the decrease of the mobility of the PLA chains, and consequently hindering the crystallization process leading to composites with lower crystallinity and higher values of *T_cc_* as well. Frone et al. [54] observed a similar behavior in PLA nanocomposites reinforced with silane treated nanofibers. 

The incorporation of lignin in the composites has also a very favorable impact on the composite thermal stability, as we can see in Figure 9 (325–385 °C). The extent of degradation is substantially reduced taking into account the value of the endothermic transition. The solid residue at 500 °C is also higher due to the formation of highly condensed aromatic structures which have the ability to form char [55]. The rise of the residue at 500 °C with the addition of lignin into PLA composites was reported in other studies [56].

**Figure 10:**
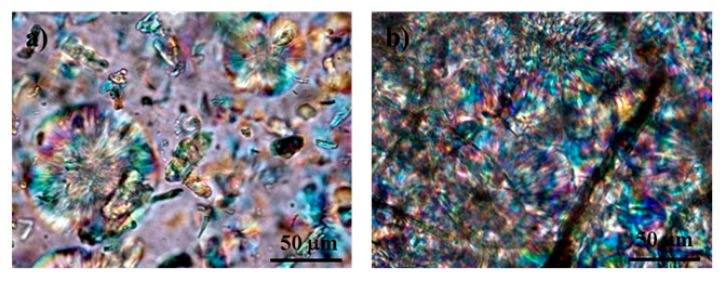
Polarized optical microscopy images of: (**a**) PLA/MCC and (**b**) PLA/UP biocomposite films (see Table 1 for sample identification).

Table 4 shows a substantial increase of the degree of crystallinity of the PLA films with the inclusion of cellulose fibers in its original dimension (from 29.2% to 54.6%). The same fibers, after being ground (GUP), which have much higher fines content, provokes a substantial decrease of the crystallinity of the PLA in the film. The additional inclusion of lignin on the fibers surface causes a huge decrease of the PLA crystallinity from 28.9% to 10.5%, in accordance with the lower mobility of the lignin adsorbed PLA molecules previously commented. The same trend occurs for the MCC, but with lower extent. Figure 10 shows that more and larger spherulites are formed in the PLA/UP biocomposite film than in the PLA/MCC. The higher specific surface area of the MCC (and grinded fibers) provides much higher nucleation points for crystallization, which hypothetically did not provide conditions for appropriate spherulites growing. On the contrary, the reduced number of nucleation points in UP fibers provides conditions for good spherulites growing, which led to very high crystallinity degree.

**Figure 11:**
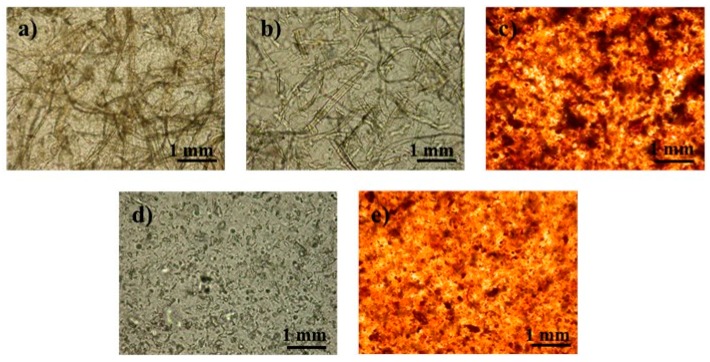
Optical microscopy images of the PLA/cellulose composite films: (**a**) PLA/UP; (**b**) PLA/GUP; (**c**) PLA/GUP-L61; (**d**) PLA/MCC; (**e**) PLA/MCC-L61 (see Table 1 for sample identification).

Structural and mechanical properties of the PLA and composite films are presented in Table 5 and Table 6, and the images of the dispersion of the cellulose into PLA matrix examined by optical microscopy are shown in Figure 11. As can be seen in Table 5, the apparent density of the films, calculated by the basis weight and thickness ratio is close to the theoretical PLA density (1.24 g/cm^3^) indicating the production of a practically porosity-free film.

Tensile strength of the composite films incorporating UP, GUP and GUP-L61 does not improve when compared with the PLA film (Table 6). In the case of PLA/UP film, the loss of tensile strength is particularly relevant and is probably due to more brittle nature of the film due to the much higher crystallinity of the PLA (Table 4). The brittle nature of the film is clearly revealed in the extremely low value of toughness determined by the integration of the stress-strain curve. In turn, the incorporation of MCC and MCC-L61 improves tensile strength and, particularly, the toughness (Table 6), due to the high increase in elongation. The relatively good dispersion of the MCC in PLA matrix (Figure 11) and the high number of interface between the crystals can tentatively explain these elongations.

The toughness performance of PLA/GUP is much higher than the PLA/UP due to the lower crystallinity of the PLA. However, it is much lower than PLA/MCC probably because of the lower elongation of the PLA/GUP composite due to the restrain imposed by cellulose fiber network.

Contrarily to observed in the present work, Mathew et al. [9] found that PLA composites with pulp fibers have better tensile properties than those with MCC. However, pulp fibers loads are higher (10–25 wt %) than that used herein. Regarding the reinforcement ability of MCC in PLA composites, the literature shows contradictory results [9,19,57,58]. Our results are in agreement with those obtained by Xian et al. [19], who observed that PLA composites with low MCC loads (2–6 wt %) improved tensile strength. According to these authors, MCC can be uniformly dispersed in PLA matrix and had good interfacial bonding with PLA, as MCC has high specific surface area, surface activity and can form hydrogen bonds with PLA [19]. 

Concerning to Young’s modulus, it was observed a decrease for the composite films when compared with PLA film, being this decrease more pronounced in the case of PLA/MCC and PLA/MCC-L61 films, in accordance with their higher elongation at break (Table 6). However, these outcomes are apparently contradictory to those reported in other studies, which found an increase of Young’s modulus and the decrease of elongation at break for PLA composites reinforced with MCC [9,19,57,58].

All composites show a decrease in the bending elastic modulus when compared with PLA film meaning that the composites are less stiff than PLA film. 

In summary, results show that PLA/MCC composite films present better mechanical properties than that of PLA/pulp fibers composite films.

## 4. Conclusions

The solvent evaporation technique was used to produce PLA/cellulose biocomposite spheres, which were processed by compression molding. It was shown that spheres yield is primarily determined by the PVA/PLA ratio, as a suitable amount of PVA must be present in the emulsion to stabilize them, preventing the droplets coalescence. As expected, the PVA/PLA ratio required increases with the decrease of spheres size. Moreover, the increase of the hydrophobic character of the cellulose fibers led to the increase of the required PVA/PLA ratio. For the optimized PVA/PLA ratio, the spheres yield ranged from 74.0% to 87.2%. The results showed the importance of cellulose surface chemistry on the spheres yield and on the percentage of cellulose incorporated. The effectiveness of cellulose fibers incorporation was improved more than 50% when the GUP pulp fibers were surface modified with 61% lignin (GUP-L61), in accordance with the increase of pulp fibers hydrophobicity. It was also shown that the reduction of the fiber size by grinding only increased cellulose incorporation from 31.4% to 36.0% confirming that the particle dimension is not the main parameter affecting cellulose incorporation. The average spheres size (weighted in volume) ranged from 130.9 to 888.0 µm for the PLA/MCC and PLA/GUP-L61 samples, respectively. The corresponding external specific surface area, determined by laser diffraction, decreased from 579.7 to 71.1 cm^2^/g. The BET specific surface area is, in general, two orders of magnitude higher, in accordance with the observed internal porosity of spheres by SEM. Despite this, practically porosity-free films were produced; the apparent density of the films is close to the density of the PLA used to produce the spheres. The incorporation of cellulose in its different forms led to a slightly decrease of the glass and melting temperature of the biocomposite films. The decrease is particularly noted in the cold crystallization temperature. On the other hand, the incorporation of UP fibers greatly increases the degree of crystallinity of the PLA. On the contrary, cellulose modified with lignin (GUP-L61 and MCC-L61) hinder the crystallization process (also noted in the significant increase in *T_cc_*) and yields biocomposite films with lower crystallinity. The mechanical properties of the biocomposite films are affected by the cellulose type. MCC improves these properties, doubling the toughness value compared with PLA film. In contrast, the incorporation of UP fibers results in substantial loss of tensile strength and toughness due to higher PLA crystallinity in the PLA/UP film. The grinded pulp fibers originate composite films with lower crystallinity and higher tensile strength and toughness compared with UP fibers.

## Figures and Tables

**Table 1:** Effect of PVA/PLA ratio on PLA/cellulose spheres yield, for different samples (cellulose can be UP: unbleached pulp, GUP—grinded UP, GUP-L25—GUP with 25 wt % lignin, GUP-L61—GUP with 61 wt % lignin, MCC—microcrystalline cellulose, MCC-L61—MCC with 61 wt % lignin).

Sample	PVA/PLA Ratio	Spheres Yield (%)
PLA	0.13	36.4 ± 4.1
PLA	0.28	85.9 ± 2.2
PLA *	0.35	93.2 ± 0.9
PLA/UP *	0.13	79.9 ± 1.2
PLA/UP	0.20	75.8 ± 1.2
PLA/GUP *	0.13	82.8 ± 3.1
PLA/GUP	0.20	76.3 ± 2.1
PLA/GUP-L25	0.28	79.6 ± 0.4
PLA/GUP-L61	0.13	7.5 ± 7.3
PLA/GUP-L61	0.20	0 ± 0
PLA/GUP-L61 *	0.28	87.2 ± 1.8
PLA/MCC	0.13	4.0 ± 1.5
PLA/MCC	0.20	4.5 ± 6.4
PLA/MCC *	0.28	74.0 ± 0.3
PLA/MCC-L61 *	0.13	84.3 ± 4.3

* Samples characterized more deeply throughout the work.

**Table 2:** Physical properties of selected samples of PLA/cellulose spheres (see Table 1 for sample identification).

Sample	Density (g/cm^3^)	Laser Diffraction			BET
		Average Spheres Size (µm)		External Surface Area (cm^2^/g)	Global Surface Area (cm^2^/g)
		Weighted by Volume	Weighted by Number		
PLA	0.78 ± 0.03	126.2 ± 1.5	46.0 ± 1.9	525.0 ± 21.2	not measured
PLA/UP	0.91 ± 0.03	796.5 ± 1.3	633.4 ± 1.3	78.3 ± 0.4	10,600
PLA/GUP	0.69 ± 0.01	611.1 ± 1.4	447.2 ± 1.3	104.5 ± 0.7	11,400
PLA/GUP-L61	0.77 ± 0.01	888.0 ± 1.4	648.4 ± 1.4	71.1 ± 0.2	9300
PLA/MCC	0.78 ± 0.00	130.9 ± 1.6	10.9 ± 1.6	579. 7 ± 11.3	5700
PLA/MCC-L61	0.83 ± 0.02	641.7 ± 1.4	305.5 ± 1.9	101.0 ± 4.5	7400

**Table 3:** Composition of the PLA/cellulose biocomposite spheres (see Table 1 for sample identification).

Sample	PLA (%)	Cellulose (%)	Lignin (%)
PLA/UP	92.8	7.2	≈ 0
PLA/GUP	92.2	7.8	≈ 0
PLA/GUP-L25	89.1	8.8	2.0
PLA/GUP-L61	82.7	7.2	10.1
PLA/MCC	95.0	5.0	0
PLA/MCC-L61	88.1	1.0	10.9

**Table 4:** DSC parameters for PLA and its biocomposite films (see Table 1 for sample identification).

Sample	*T_g_* (°C)	*T_cc_* (°C)	Δ*H_cc_* (J/g)	*T_m_* (°C)	Δ*H_m_* (J/g)	*X_c_* (%)	*T_d_* (°C)	Solid Residue (%)
PLA	62.1	96.1	32.72	177.7	60.04	29.2	368.2	1.2
PLA/UP	60.0	90.7	7.09	175.2	54.49	54.6	359.4	2.0
PLA/GUP	58.6	92.7	26.58	174.8	51.54	28.9	365.7	1.2
PLA/GUP-L61	58.7	108.9	37.04	174.3	45.13	10.5	358.9	10.0
PLA/MCC	61.3	93.8	29.00	176.7	55.09	29.3	368.3	1.0
PLA/MCC-L61	62.1	100.8	34.07	175.9	53.88	24.0	357.1	5.4

**Table 5:** Structural properties of PLA/cellulose biocomposite films (see Table 1 for sample identification).

Sample	Basis Weight (g/m^2^)	Thickness (µm)	Apparent Density (g/cm^3^)
PLA	153.4 ± 8.2	127.8 ± 10.2	1.205 ± 0.087
PLA/UP	163.3 ± 13.3	134.0 ± 12.4	1.220 ± 0.039
PLA/GUP	152.8 ± 10.5	125.7 ± 11.0	1.218 ± 0.037
PLA/GUP-L61	153.5 ± 7.9	126.3 ± 11.1	1.223 ± 0.122
PLA/MCC	161.9 ± 8.0	132.7 ± 11.6	1.224 ± 0.054
PLA/MCC-L61	154.4 ± 2.8	127.0 ± 4.4	1.217 ± 0.020

**Table 6:** Mechanical properties of PLA/cellulose biocomposite films (see Table 1 for sample identification).

Sample	Tensile Strength (MPa)	Elongation at Break (%)	Young’s Modulus (GPa)	Toughness (kJ/m^3^)	Bending Elastic Modulus (MPa)
PLA	35.1 ± 5.0	2.7 ± 0.4	1.82 ± 0.27	520 ± 78	4.45 ± 0.46
PLA/UP	23.4 ± 3.6	1.5 ± 0.2	1.65 ± 0.26	211 ± 49	4.15 ± 1.01
PLA/GUP	30.6 ± 6.8	2.2 ± 0.3	1.66 ± 0.16	383 ± 149	3.83 ± 0.63
PLA/GUP-L61	35.6 ± 6.1	2.3 ± 0.4	1.66 ± 0.09	408 ± 125	4.34 ± 1.13
PLA/MCC	43.0 ± 3.4	3.6 ± 0.7	1.53 ± 0.18	937 ± 267	4.07 ± 0.79
PLA/MCC-L61	44.9 ± 2.4	3.8 ± 0.3	1.55 ± 0.09	970 ± 129	4.36 ± 0.42
